# Heart failure and late-onset Alzheimer’s disease: A Mendelian randomization study

**DOI:** 10.3389/fgene.2022.1015674

**Published:** 2022-11-29

**Authors:** Yibeltal Arega, Yongzhao Shao

**Affiliations:** Department of Population Health, New York University Grossman School of Medicine, New York, NY, United States

**Keywords:** Alzheimer’s disease, heart failure, causal association, vascular dementia, Mendelian randomization, SNPs, GWAS

## Abstract

Some observational studies suggested that heart failure (HF) is associated with increased risk of late-onset Alzheimer’s disease (AD). On the other hand, a recently published Two-Sample Mendelian Randomization (2SMR) study was reported as inconclusive but the estimated odds ratios (ORs) were less than one indicating a potential causal association between genetically predicted HF and lowered risk of AD. Both HF and AD are quite common among elderly persons and frequently occur together resulting in a series of severe medical challenges and increased financial burden on healthcare. It is of great medical and financial interest to further investigate the statistical significance of the potential causal associations between genetically predicted HF and lowered risk of AD using large independent cohorts. To fill this important knowledge gap, the present study used the 2SMR method based on summary data from a recently published large genome-wide association study (GWAS) for AD on subjects with European ancestry. The 2SMR analysis provided statistically significant evidence of an association with ORs less than one between genetically predicted HF and late-onset AD (Inverse Variance Weighted, OR = 0.752, *p* = 0.004; MR Egger, OR = 0.546, *p* = 0.100; Weighted Median, OR = 0.757, *p* = 0.014). Further investigations of the significant associations between HF and late-onset AD, including specific genes related to the potential protective effect of HF-related medications on cognitive decline, are warranted.

## Introduction

Alzheimer’s disease (AD) is the most common progressive neurodegenerative disease. Currently, there is no cure for AD. The vast majority of Alzheimer’s dementia occur among people age 65 or older, called late-onset AD. AD has heterogeneous etiology. Advanced age, family history of AD, diabetes, hypertension, and some lifestyle factors are among the known major risk factors (Gaugler et al., 2022). According to the World Alzheimer Report 2016, there were about 46.8 million people who have AD worldwide. The number of AD patients is expected to double nearly every 20 years, and thereby the AD population is expected to reach 74.7 million or more in 2030 and 131.5 million in 2050 (Du et al., 2018). AD patients show progressive memory loss, gradual impairment of cognitive functions, and possible behavioral and personality changes. AD severely burdens patients’ families and healthcare systems (Ohno et al., 2021).

Heart failure (HF) has an increasing prevalence and affects about 26 million people worldwide and 5.7 million people in the USA a decade ago and will increase with an aging population. The burden of HF-related hospitalizations and costs is increasing. In 2012, the total healthcare costs for HF were estimated to be about US $30.7 billion; by 2030, projections suggest an increase of 127% (Savarese et al., 2022). HF and AD share multiple known common risk factors, such as lifestyle, air pollution, and others. For example, advanced age, diabetes mellitus, hypertension, obesity, and smoking all play major roles in the development of HF as well as AD (Gottesman et al., 2017). In particular, existing epidemiological and experimental studies demonstrated that air pollution also increases the risk of both cardiovascular disease and cognitive decline (Cohen et al., 2017; Clouston et al., 2022; Rosen et al., 2022).

Heart failure and dementia frequently occur among elderly persons due to many shared confounding risk factors and thus result in a series of severe medical and financial problems. Impaired cognition in HF patients can lead to more frequent hospital readmissions and increases mortality rates. AD and vascular dementia are the two most common types of dementias, it is of interest to know whether HF is a major risk factor for vascular dementia or AD, or for both of them. Therefore, this highlights the need to study HF, vascular dementia and AD as well as their relationships. Indeed, the association between heart failure (HF) and late-onset Alzheimer’s disease (AD) has been investigated in many populations, however, the reported findings are inconsistent. It has been suggested that HF is a risk factor for AD by Qiu et al. (2006) and multiple other reports as reviewed by Cermakova et al. (2015a). As is well known, findings from observational studies are often biased due to failures to adequately control for both measured and unmeasured confounders. The effect estimates from observational studies are often exposed to certain deficiencies, thus, they are often biased and lack of consistency in different studies and publications. More recently, Li et al. (2020) did not find evidence to show a significant association between HF and AD using meta-analysis. Moreover, Adelborg et al. (2017) conducted a large national-wide population-based cohort study in Denmark on the association between HF, vascular dementia, and AD. In their published report [e.g., Table 4 of Adelborg et al. (2017)], the hazard ratios (HRs) indicate that HF is significantly associated with increased risk of vascular dementia and other non-AD dementias regardless of follow-up years with hazard ratios (HRs) significantly larger than one (HRs > 1). This is consistent with reports of many other studies on HF being risk factor for all-cause dementia. However, regarding AD, the HRs and their 95% confidence intervals are clearly all below one (HRs < 1) indicating that HF is significantly associated with lowered risk of AD if the patients were followed 11–35 years. To date, this large population-based study of more than 300,000 HF subjects and more than 1,600,000 non-HF population controls with more than 30 years of follow up is the largest known well-designed study on HF, vascular dementia, and AD. The data from this study (e.g., Table 4) indicated HF is a significant risk factor of vascular dementia but not AD. In fact, in long-term follow-ups (11–35 years), HF is associated with significantly lowered risk for AD.

Given the inconsistent reports from existing observational studies, it is desired to have some causal-inference based studies that are robust to the influence of confounders. Exploring causal associations between HF and AD is essential for researchers to pursue new treatment strategies or to obtain a relatively accurate prognosis for patients (Duan et al., 2022). Mendelian Randomization (MR) is increasingly being used to study causality. MR is an approach that uses genetic variants to assess causal relationships between exposures or risk factors and subsequent clinical outcomes. The main idea of MR is that genetic alleles are randomly allocated at birth, thus, frequently, there is no influence from confounders on the predisposed genetic variants (Sanderson et al., 2022). In particular, the Two-Sample Mendelian Randomization (2SMR) approach can estimate the causal relationship between exposure and outcome using the summary datasets of genome-wide association study in the absence of individual-level data on genetic variants.

Duan et al. (2022) applied the 2SMR method to test the causal relationship between genetically predicted HF and AD. They did not report a significant causal relationship between genetically predicted HF and AD. However, the estimated odds ratios (ORs) were all less than one indicating a potential association of HF with lowered risk of AD. The MR studies measure the lifetime risk of genetically predicted exposures and subsequent disease outcomes. The finding of ORs <1 in this 2SMR study is consistent with the finding of HRs <1 reported in Table 4 of the largest population-based study with long-term follow-up (Adelborg et al., 2017). Duan et al. (2022) did not report the finding as statistically significant but stated that “more large-scale datasets are expected for further MR analysis” possibly reflecting concerns that the statistical power of study might be low as the GWAS dataset for AD used might not be large enough.

**FIGURE 1:**
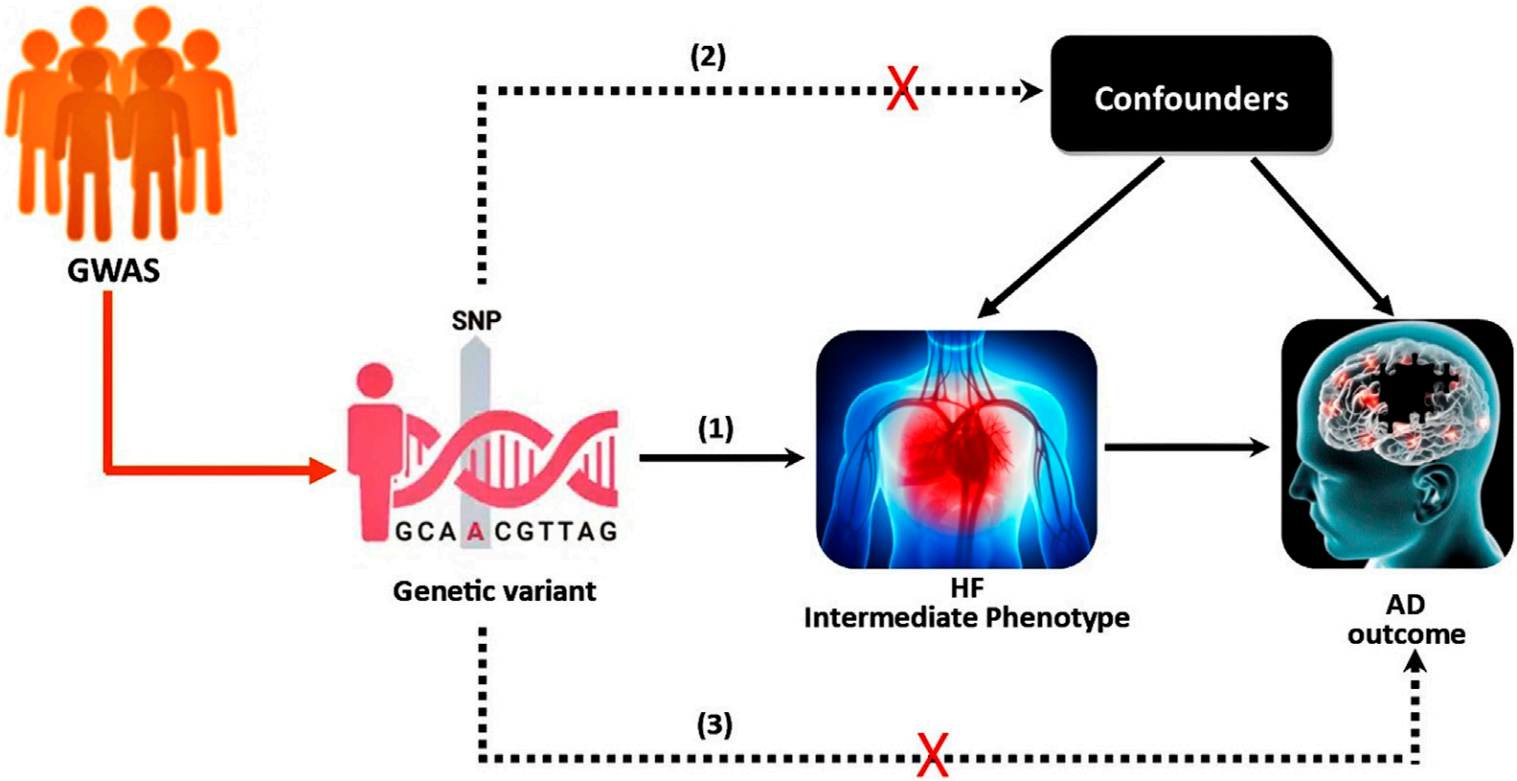
The instrumental variable (IV) assumptions for MR. (1) The genetic variant must be associated with the intermediate phenotype (exposure); (2) the genetic variant should not be associated with confounders; (3) the genetic variant must not influence the outcome except through the exposure. The dashed lines represent pathways that violate the assumptions.

Based on the findings of the largest well-designed population-based study (Adelborg et al., 2017) and the new 2SMR-based causal analysis (Duan et al., 2022), HF is clearly a significant risk factor for all-cause dementia, particularly, a major risk factor of vascular dementia. However, there is no convincing evidence that HF is a causal risk factor of AD. In fact, the HRs and ORs of HF on AD are both less than one in Adelborg et al. (2017) and Duan et al. (2022), respectively. We hypothesize that the reported HRs and ORs (being less than one) are valid and reproducible when further investigated using large independent cohorts. We speculated the ORs (being less than one) reflect the protective effect of HF-related medications, survival bias, and possibly some shared genetic mechanism. In general, heart failure is commonly treated with HF-related medications and many of them have been reported to have protective effects on cognitive decline and AD (Barthold et al., 2020; Poly et al., 2020; Nguyen et al., 2022; Olmastroni et al., 2022). Additionally, it is widely reported that AD pathology might begin 20 years before the clinical diagnosis of AD (e.g., Figure 1; Golde, 2022). Thus, patients of HF may have less chance to develop late-onset AD because HF is a leading cause of short survival in many western populations. Identification and characterization of further biological mechanisms that underlying the lowered risk of AD among HF patients are among the important further research topics.

In short, both HF and AD are quite common among elderly persons, causing a series of severe medical challenges and increased financial burden. Currently there is no cure for AD and no effective prevention methods. Establishing a significant causal association between HF-related medications and late-onset AD might facilitate the development of effective preventive measures for AD *via* exploring suitable combinations of commonly used medications for HF. Therefore, it is of great medical and financial interest to further investigate the statistical significance and causality of the associations between HF and the risk of AD using large independent cohorts. To fill these important knowledge gaps, the present study conducted a 2SMR-based causal analysis to test the novel hypothesis that genetically predicted HF is causally associated with lowered risk of AD based on publicly available summary data of GWAS on HF (Shah et al., 2020) and summary data of a new and large GWAS on AD (Bellenguez et al., 2022).

## Materials and methods

The 2SMR was developed to assess the causal effect of exposure or intermediate phenotypes on the final disease outcome, and it uses independent samples from the same population or comparable populations to assess gene-exposure and gene-outcome associations (Hemani et al., 2018). The validity of the 2SMR method requires three basic assumptions; 1) the genetic variants are associated with the intermediate phenotype (exposure); 2) genetic variants are independent of the confounders; and 3) genetic variants do not have a direct effect on the disease (outcome) or no horizontal pleiotropy (Davies et al., 2018). Graphically, the study scheme of 2SMR is demonstrated in Figure 1. Recently, Duan et al. (2022) studied the potential causal association between HF and AD using the 2SMR method, but did not report any significant findings. In their 2SMR study, there are some concerns about statistical power. In particular, there are concerns about the heterogeneity among the nine selected instrumental variables (IVs), and some of the nine candidate SNPs’ might have disproportionately large influence. Similar to Duan et al. (2022), we also employed 2SMR to study the causal relationship between HF as the intermediate phenotype and AD as the final outcome using a much larger GWAS data.

### Data source

This study used a 2SMR approach based on publicly available summary-level GWAS data, as summarized in Table 1. We used the summary data of a recently published GWAS on AD (Bellenguez et al., 2022) derived from the European Alzheimer and Dementia Biobank (EADB) consortium. It consists of 487,511 (85,934 cases and 401,577 controls) individuals of European ancestry. The GWAS on HF data was originally from the Cardiovascular Disease Knowledge Portal (CVDKP) which contained 977,323 (47,309 cases and 930,014 controls) individuals of European ancestry (Shah et al., 2020). The AD and HF GWAS summary datasets are available in the GWAS catalog (https://www.ebi.ac.uk/gwas/). The web sources are provided in Table 1.

**TABLE 1:** Details of GWAS datasets used for the 2SMR analysis.

Exposure/Outcome	Consortium/Publication	Participants	Web source
HF	CVDKP (Shah et al., 2020)	977,323 individuals of European ancestry (47,309 cases and 930,014 controls)	https://www.ebi.ac.uk/gwas/studies/GCST009541
AD	EADB (Bellenguez et al., 2022)	487,511 individuals of European ancestry (85,934 cases and 401,577 controls)	https://www.ebi.ac.uk/gwas/studies/GCST90027158

### Selection of instruments and MR analysis

The Mendelian randomization method can be viewed as an instrumental variable approach to causal inference. To identify strong instrumental variables (IVs), we selected SNPs that were associated with the exposure (HF) at a genome-wide significant threshold for *p*-values ( $p<5\times 10^{-8}$ ) in the available GWAS summary data of the HF study published by Shah et al. (2020). Similar to Duan et al. (2022), we checked SNPs that were in linkage disequilibrium (LD), which indicates the association between alleles at the linked loci (within a physical distance of 10,000 kb and $r^{2}<0.001$ ). Using PhenoScanner (Kamat et al., 2019), we checked a possible direct association of a SNP with AD. The corresponding SNPs associated with AD were searched in the AD GWAS dataset. If a particular SNP is not present in the AD dataset, then it is possible to use SNPs that are LD proxies instead. The proxy SNPs are in high LD (as a threshold, $r^{2}>0.8$ ). Subsequently, data harmonization was conducted. In the data harmonization step, the effect direction was performed to ensure that SNPs’ effect on HF and AD corresponded to the same allele (Hemani et al., 2018). The strength of association between SNPs and exposure (HF) was measured with F statistic, $F=\beta^{2}/{SE}^{2}$ , where β and SE are estimated exposure effect and standard error, respectively (Bowden et al., 2016). We extracted the retained SNPs from the outcome datasets and tested the causal relationship between HF and AD using 2SMR. The causal effect was assessed using four different 2SMR estimation methods: Inverse Variance Weighted (IVW), MR-Egger, weighted median and weighted mode. The heterogeneity across genetic variants was evaluated using the Cochran Q test. The presence of pleiotropy was evaluated using the intercept of the MR-Egger regression method (Bowden et al., 2015; Hemani et al., 2018). Furthermore, a leave-one-out analysis was used to check the outlier or sensitive genetic variants, which is used to test whether there is an SNP that has a disproportional impact on MR estimates. In the leave-one-out plot, if the estimated effect changes noticeably when one of the SNP is removed, its effect was considered as disproportionate (Burgess and Thompson, 2017). All statistical analysis was conducted using R (version 4.1.3) and “TwoSampleMR” R package (version 0.5.6).

## Results

As initial analysis, after quality assurance and LD-based selection step, this new study started with the same set of candidate instrumental variables as in Duan et al. (2022). In particular, 12 SNPs are associated with HF at a genome-wide significant threshold ( $p<5\times 10^{-8}$ ) without evidence of LD ( $r^{2}<0.001$ within 10,000 kb physical distance). Each SNP was checked with potential pleiotropic effects using the PhenoScanner tool, and there are no SNPs that were found to be associated with the possible direct mechanistic pathways of AD. During data harmonization, three SNPs (rs1556516, rs4746140, rs4766578) were removed from the candidate set of 12 SNPs for being palindromic with intermediate allele frequencies. After data harmonization, nine SNPs were considered as potential instrumental variables for the 2SMR test. The strength of association between SNPs and HF was measured with the F statistic values ranging from 30.89 to 83.10. These values indicated no weak instrumental effects or all are above the threshold value (F>10) as recommended in Bowden et al. (2016). The detailed potential IVs are presented in Table 2 and Supplementary Table S1.

**TABLE 2:** The five candidate SNPs as instrumental variables (IVs) for MR analysis of the causal effect of HF on AD.

SNP	Chr	Pos	Gene	A1	A2	EAF	Beta	SE	pval	F
rs1510226	6	160816409	SLC22A3	C	T	0.01	0.162	0.029	1.27E-08	32.31
rs17617337	10	121426884	BAG3	T	C	0.22	−0.056	0.010	3.65E-09	34.87
rs4135240	6	36647680	CDKN1A	C	T	0.34	−0.049	0.008	6.84E-09	33.47
rs55730499	6	161005610	LPA	T	C	0.07	0.106	0.016	1.83E-11	45.41
rs600038	9	136151806	ABO, SURF1	C	T	0.21	0.057	0.010	3.68E-09	35.13

SNP, single-nucleotide polymorphism; Chr, chromosome; Pos, position; A1, effect allele; A2, non-effect allele; EAF, effect allele frequency; Beta, beta estimate for the association of SNP with HF; SE, standard error; pval, *p*-value from the meta-analysis of HF.

Duan et al. (2022) used the GWAS on AD dataset reported in Kunkle et al. (2019) and did not report any finding of significant evidence suggesting a causal relationship between genetically predicted HF and a risk of AD. However, the odds ratios for the causal effect of the exposure HF on AD outcome were all less than one (OR < 1). The lack of strong evidence of statistical significance of the estimated odds ratios might be because of a not large enough GWAS dataset on AD and there might be heterogeneity with some candidate SNPs having disproportionate influence. To overcome this potential limitation, we used a new and larger AD GWAS summary dataset reported in Bellenguez et al. (2022) with a sample size more than seven times of the GWAS on AD reported in Kunkle et al. (2019). In particular, the GWAS reported in Bellenguez et al. (2022) used a larger sample (487,511 individuals of European ancestry) while the GWAS on AD reported in Kunkle et al. (2019) studied 63,926 individuals of European ancestry.

**FIGURE 2:**
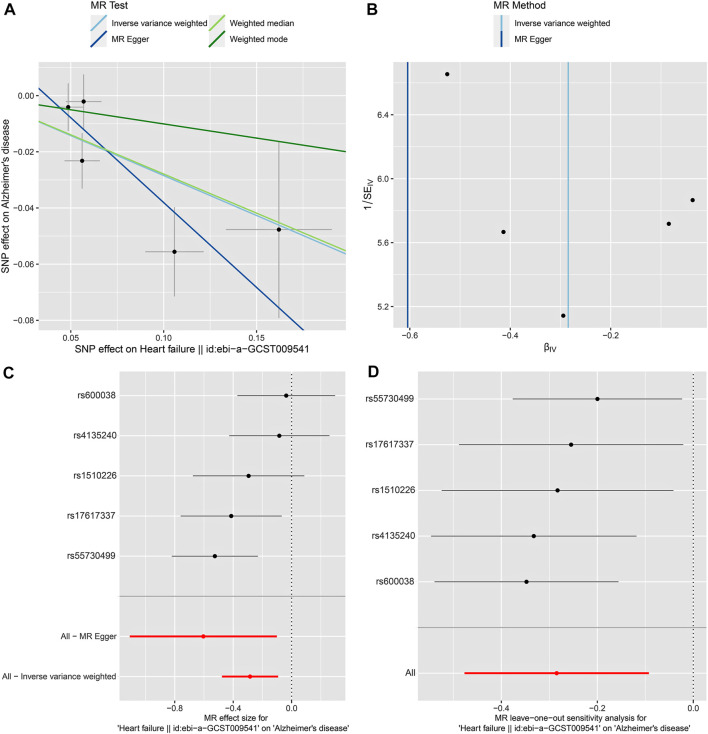
2SMR analysis of the causal association between HF as exposure and AD as disease outcome using the five selected SNPs and the most recent AD GWAS dataset (Bellenguez et al., 2022). **(A)** Scatter plot. Each point represents an instrumental variable (SNP). The colored lines correspond to the different methods of causal estimate, and the horizontal and vertical lines crossing at each point show 95% CI for each SNP. **(B)** Funnel plot. The vertical line shows a causal estimate using all SNPs combined into a single instrument for two different methods. **(C)** Forest plot. Each black dot represents the MR estimate of each SNP, and the horizontal line represents the 95% CI. The red points show a combined causal estimate using all SNPs, including the 2SMR estimates of IVW and MR-Egger. **(D)** Leave-one-out sensitivity analysis. Each black dot represents the estimate of MR-IVW excluding that particular SNP, and the associated horizontal line depict 95% CI. The red dot depicts the IVW estimate using all SNPs.

In our 2SMR analysis, we were able to reproduce the Duan et al. (2022) 2SMR results including Table 2 and Table 4 of Duan et al. (2022) using exactly the same datasets they used. We carefully examined the effect of each SNP using a leave-one-out sensitivity analysis. The odds ratios are indeed all smaller than one (OR < 1) indicating that genetically predicted HF is potentially associated with lowered risk of AD. Typically the inverse variance weighted (IVW) method is quite powerful in the absence of heterogeneity among the candidate SNPs satisfying the three IV assumptions. The fact that the weighted median method had a *p*-value *p* = 0.025 < 0.05 indicates potential causal association between HF and AD [Table 2 of Duan et al. (2022)]. The inverse variance weighting method has a slightly weaker *p*-value (*p* = 0.088) indicating a possibly loss of power due to heterogeneity among IVs. Our analysis indicates that potentially some of the nine IV SNPs may have large disproportional influence on the MR estimate [see Table 2 of Duan et al. (2022)]. We conducted the leave-one-out sensitivity analysis and found the SNP rs17042102 potentially has large influence, see Supplementary Figure S1D. In general, meta-analysis results in the presence of heterogeneity and/or some SNPs with extremely large influence may not be reproducible in independent datasets even with larger sample sizes. The heterogeneity Cochran Q-test for meta-analysis may become statistically significant under larger sample sizes of the new independent dataset. Indeed, our analysis indicates that Q-test for heterogeneity is significant (*p* < 0.05) for the nine SNPs in the large dataset (Bellenguez et al., 2022). Given the weighted median method provided a significant MR estimate, a suitable subset of homogeneous SNPs among the nine SNPs may yield results more likely to be generalizable and reproducible under independent large datasets and may pass the Cochran Q-test under large sample sizes. Subsequently, from the 9 SNPs, we identified that the subset of five SNPs, rs55730499, rs4135240, rs17617337, rs600038 and rs1510226. These five SNPs had stable impact on the 2SMR test results (Supplementary Figure S1D). The five SNPs all located in the middle of the plot in Supplementary Figure S1D with Cochran Q-test *p*-value > 0.94 indicating no-evidence of heterogeneity (Supplementary Table S2). Using these five SNPs, we recomputed the 2SMR test on the datasets used by Duan et al. (2022). We did find evidence that all the odds ratios are still similar to the published ORs identified by Duan et al. (2022) indicating stability and potentially reproducible association between HF and AD using independent datasets with larger sample sizes (IVW, OR = 0.774, *p* = 0.070; MR Egger, OR = 0.923, *p* = 0.859; weighted median, OR = 0.745, *p* = 0.094; weighted mode, OR = 0.729, *p* = 0.243) (Supplementary Table S2). To ensure adequate statistical power, we used the larger AD GWAS datasets (Bellenguez et al., 2022) and re-tested the potential causal association between HF and AD using the five SNPs. Indeed, with the new and larger AD GWAS dataset, the 2SMR summary test results provided clear evidence of a statistically significant causal association between genetically derived HF and lowered risk of late-onset AD in both IVW and weighted median 2SMR methods (IVW, OR = 0.752, *p* = 0.004; MR-Egger, OR = 0.546, *p* = 0.100; weighted median, OR = 0.757, *p* = 0.014; weighted mode, OR = 0.904, *p* = 0.639). As expected, both the IVW and weighted median method yield significant ORs. The directions of our estimated odds ratios of the five SNPs are consistent with those published in Table 2 of Duan et al. (2022), i.e., ORs < 1 (Table 3). The heterogeneity and horizontal pleiotropy tests were assessed using the Cochran Q test and MR Egger intercept test, respectively. The Cochran Q test for meta-analysis showed no evidence of significant heterogeneity (IVW *p* = 0.163, and MR Egger *p* = 0.250), and the MR-Egger intercept suggested no evidence of significant horizontal pleiotropy even with the very large sample sizes (MR Egger intercept *p* = 0.280) (Table 3). Likewise, using the small datasets used in Duan et al. (2022) provides the same sensitivity analysis results for these five SNPs (Supplementary Table S2). In addition, we checked the effect of each SNP’s influence on the causal estimate test using graphical methods. The leave-one-out sensitivity analysis was used to test the undue influence of potentially pleiotropic SNPs on the causal estimates. We did not find any SNP among the five selected SNPs that disproportionately impacted the results. A Funnel plot was generated to visually evaluate the horizontal pleiotropy. Asymmetry in the funnel plot indicative of horizontal pleiotropy. Moreover, the scatter plot shows the association between SNPs on the HF versus SNPs on the AD, whereas, forest plot shows result to evaluate potential causal associations between HF and AD using a single SNP at a time (Figure 2; Supplementary Figure S2). Finally, we compared the power of our dataset with Duan et al. (2022) dataset using online MR power calculation tool (https://sb452.shinyapps.io/power/) (Burgess, 2014). As input, we used IVW estimates, and computed the coefficient of determination (*R* ^2^) of exposure on genetic variants using the TwoSampleMR R package. Our dataset reached 97.9% power and Duan et al. (2022) dataset reached 36.1% power. Therefore, our dataset reached a much higher power to detect a causal effect between HF and AD. In short, the above findings showed a statistically significant causal association between genetically predicted HF and a lower risk of late-onset AD.

**TABLE 3:** 2SMR estimates of the causality between the exposure HF and the AD outcome.

Methods	nSNPs	OR (95%CI)	*p*-value	Q pval	Intercept pval
IVW	5	0.752 (0.621, 0.912)	0.004	0.163	
MR Egger	5	0.546 (0.330, 0.904)	0.100	0.250	0.280
Weighted median	5	0.757 (0.606, 0.945)	0.014		
Weighted mode	5	0.904 (0.613, 1.335)	0.639		

nSNPs, number of SNPs; OR, odds ratio; CI, confidence interval; Q pval, *p*-value of the Cochran Q test; IVW, inverse-variance weighted; Intercept pval, *p*-value of MR-Egger intercept test.

## Discussion

HF and AD frequently occur among elderly persons and are known for sharing many common risk factors. There have been many population-based observational studies that reported high prevalence of both diseases and positive association between them. However, positive association between HF and AD can simply be due to the fact that both share common lifestyle and other risk factors such as obesity, diabetes mellitus, smoking, and hypertension (Gottesman et al., 2017). For example, some studies reported that obesity is a major modifiable factors that increases the risk of HF (Lavie et al., 2016) and AD (Slomski, 2022). Likewise, diabetes mellitus is also a lifestyle-related risk factor for both HF and AD (Rosano et al., 2017; Lee et al., 2018). In addition to lifestyle confounding factors, environmental exposure is an important but often underappreciated risk factor contributing to the development and severity of cardiovascular diseases, cognitive impairment, and late-onset AD, particularly among elderly persons. For example, Escolar et al. (2019) reported that major air pollutants, such as SO_2_ (precursor of acid rain) (r = 0.2692, *p* < 0.001) and NOX air (major air pollutant formed by combustion systems and motor vehicles) (r = 0.2196, *p* < 0.001) have a significant impact on the HF-related hospital admissions rate and, hence, on HF decompensations and patient’s quality of life. In addition to many published associations between World Trade Center (WTC) related environmental exposures and cardiovascular symptoms, Clouston et al. (2022) and Rosen et al. (2022) reviewed a large literature of published studies on significant associations of WTC-related exposures and potentially AD-related cognitive impairment.

The relationship between HF and late-onset AD has been investigated using observational studies in the last two decades. In general, the reported findings are not consistent due to well-known factors including different study design, different population characteristics, different sample sizes, and different ways to control confounding factors. To date, the largest population-based study included 324,418 HF patients with a first-time hospitalization for HF and 1,622,079 matched control individuals in the same Danish population. More than 30 years of individual-level data (between 1980 and 2012) from Danish medical registries were linked in this nationwide population-based cohort study comparing HF patients with a sex-, year of birth-, and calendar year-matched comparison cohort from the Danish population. Hazard ratios (HRs) for the risk of all-cause dementia, Alzheimer’s disease, vascular dementia, and other dementias were computed using Cox regression stratified by age, sex, follow-up time, and comorbidity and considering death as a competing risk. Based on the published findings of this well-designed population-based study (Adelborg et al., 2017), HF is clearly a significant risk factor for all-cause dementia (adjusted HR = 1.21, 95% CI = 1.18–1.24), particularly, HF is a strong risk factor for vascular dementia (adjusted HR = 1.49, 95% CI = 1.40–1.59). In contrast, they reported no evidence indicating HF being a risk factor of AD. In fact, the HRs of HF on vascular dementia are all significantly greater than one, however, the HRs of HF on AD are less than one in long-term follow-up (11–35 years) for older people (adjusted HR = 0.69, 95% CI = 0.56–0.85, for 70–79 years; adjusted HR = 0.35, 95% CI = 0.13–0.92, for ≥80 years) [Table 3 of Adelborg et al. (2017)]. In addition, for subjects without one of the seven common comorbid conditions of HF, the reported HRs ranged from 0.84 to 0.87 and all the associated 95% CIs lie below one regardless of age [Table 4 of Adelborg et al. (2017)]. These less-than-one HRs and their CIs imply that HF is significantly associated with lowered long-term risk for AD compared to matched non-HF population controls. These HRs being less than one (HRs < 1) are under-reported in literature, nevertheless, they are likely valid and reproducible findings given the solid population-based study design with vast sample sizes for both HF cases and matched populations controls.

There are many inconsistent reports from observational studies where some reported evidence that HF is a risk factor of AD while others reported no significant evidence indicating that HF is associated with AD. This inconsistency among different reports is related to different study designs, variation in sample sizes, potential heterogeneity in HF-related treatments between study populations, different length of follow-up from first diagnosis of HF to the first onset of AD, and other uncertainties/biases related to unobserved confounders. As is well known, in observational studies, the distribution of important confounders may not be the same across studies and these confounders may not be measured in the same way across different studies because of common limitations on cost, study duration, and various other practical constraints. Importantly, the inconsistency between studies may also reflect heterogeneous quality in study designs and different levels of rigor in data analysis. For example, among the observational studies, the finding for association between HF and AD reported in Qiu et al. (2006) is among the most widely cited. However, it is straightforward to identify multiple potentially major limitations in study designs, case status ascertainment, defects in data analysis and other sources of potentially major biases in Qiu et al. (2006) and many similar observational studies. Specifically, first, Qiu et al. (2006) used time-to-event analysis but did not identify the length of time since the first diagnosis of HF to the study baseline. A clear definition of time-zero and consistently applying it to all subjects are among the basic requirements for survival analysis to avoid biased findings. Thus, the years from the first diagnosis of HF to the onset of AD is calculated with inconsistency and uncertainty. That is a source of possible severe bias in the computation of HRs. Second, Qiu et al. (2006) did not consider vascular dementia as a possible outcome in the study and a large portion of the vascular dementia cases might have been misclassified as AD. HF is strongly associated with an increased risk for vascular dementia, and the adjusted incidence ratio of vascular dementia can be higher than AD among HF patients. In fact, a later publication on heart failure and dementia for the same Swedish Heart Failure Registry linked with the Swedish Dementia Registry by Cermakova et al. (2015b) reported that vascular dementia was indeed the most common dementia disorder (36%) and Alzheimer disease was the least common type of dementia (16%) behind two other non-AD dementia types. Thus, potentially major biases in the estimation of HRs may have occurred due to potential misclassification errors of AD and vascular dementia and other dementias. Third, Qiu et al. (2006) did not specify a cut-off time to define incidental AD in their time-to-event analysis. It is widely reported that it can take more than 20 years from the start of AD pathology to the clinical diagnosis of AD dementia [e.g., Figure 1; Golde (2022)]. Most incidental AD cases in the study within a very short period of follow-up after diagnosis of the first HF were likely not caused by HF or might be a non-AD dementia. The ascertainment of HF status in the study was also bias-prone. All the participants were very old at the baseline (age ≥ 75 years). The incidence rate of HF is known to be quite high among elderly persons. Importantly, more than 40% of the elderly control participants were known to have hypertension at baseline. Hypertension is a major risk factor for HF, thus many of them would likely to have developed HF during the study follow-up. However, none of the incidental HF cases were counted as having HF, instead, they were all counted as non-HF controls. Given the misclassification errors of both incidental HF and incidental AD in the study and the relatively short follow-up time (mean follow-up 5.02 years per person), the number of HF-related AD may be severely over-estimated, and potentially serious biases in the estimation of HRs might have occurred. Fourth, survivor-bias was not properly adjusted in the data analysis. As is well known, HF is a leading cause of death among elderly persons and the probability of death after HF before developing any dementias is non-negligibly high with median survival less than 4 years as reported in Cermakova et al. (2015b). When computing incidence rate and follow-up time, Qiu et al. (2006) defined censored time of follow-up as from baseline to last follow-up or death. Observed deaths should not be simply treated as censored because dead subjects will have zero probability to develop incidental AD. Death should be treated as a competing risk instead of censored. The definition of follow-up time for subjects with event was also problematic given the long follow-up period. Instead, cumulative incidence functions in competing risk would have been more suitable to avoid the biases. Most observational studies [except Adelborg et al. (2017)] did not use competing risk models and did not properly adjust for survivor-bias in their analyses of the association between HF and AD in published reports. Unfortunately, many meta analyses on the association between HF and AD published in subsequent years were also adversely affected by these biased reports. Fifth, the sample size of Qiu et al. (2006) is very small compared to Adelborg et al. (2017). In particular, they only considered 205 baseline HF cases [compared to >300,000 HF cases studied in Adelberg et al. (2017)]. Qiu et al. (2006) only considered 1,096 controls which is a small number compared to the more than 1,600,000 controls studied in Adelberg et al. (2017). No stratified analyses based on age, sex, years of follow-up were conducted due to small sample sizes in Qiu et al. (2006). The number of unmeasured confounders (years since diagnosis of HF, incidental HF status, vascular dementia status, mis-specified AD status) and latent sources of biases and uncertainties (misclassifications of AD, misclassifications of HF, uncertainty in indetermination of the length of time between AD diagnosis and study baseline) associated with the estimation of HRs and CIs is quite large relative to the sample size. These biases and uncertainties are unlikely to cancel or balance each other out. In short, due to the small sample sizes, questionable ascertainment of case and control status, questionable definition of time-zero in the time-to-event based analysis, failure to adjust for survivor-bias and competing risk, the resulting HRs from such analyses as in Qiu et al. (2006) and many other similar observational studies might be severely biased and might yield misleading results contradicting to those well-designed studies such as Adelborg et al. (2017).

In general, observational studies only establish association, not causality, no matter how large the sample sizes are. Findings of shared common risk factors for both HF and AD from observational studies might be useful because controlling the shared confounding risk factors might lead to reduction of population-level incidence for both HF and AD. However, inconsistency of study findings, potential biases due to incompletely measured confounders, and lack of evidence of causality often severely limit the utility of findings from these observational studies. Non-causal factors typically are not useful targets for developing effective disease intervention or disease prevention procedures, thus, formal causal inference methods are needed in order to determine whether HF could be a causal risk factor for AD. Mendelian randomization (MR) is a well-known causal inference method that is generally robust against both measured and unmeasured confounders. Specifically, MR uses selected germline genetic variants as instrumental variables (IVs) to assess causal relationships between IV-predicted exposures and subsequent disease outcomes. The basic idea of MR is that genetic alleles are randomly allocated at birth. Thus, there is generally no significant influence from confounders on the predisposed genetic variants (Sanderson et al., 2022). In particular, the Two-Sample Mendelian Randomization (2SMR) method is often used to assess the causal relationship between a genetically predicted exposure and a disease outcome using summary datasets from two independent genome-wide association studies in the absence of individual-level data on genetic variants.

Recently, Duan et al. (2022) applied the 2SMR method to test the causal relationship between genetically predicted HF and AD. They used summary data of a GWAS on HF reported in Shah et al. (2020) and summary data of a GWAS on AD reported in Kunkle et al. (2019). They did not report a statistically-significant causal relationship between genetically predicted HF and AD. However, the estimated odds ratios were all less than one indicating a potential causal association of HF with a lowered risk of AD. The finding of ORs <1 in this 2SMR study is consistent with the finding of HRs <1 reported in Table 4 of the largest population-based association study (Adelborg et al., 2017). Although Duan et al. (2022) did not report the finding as statistically significant, they stated that “more large-scale datasets are expected for further MR analysis” due to concerns that the statistical power of study might be low as the GWAS dataset for AD used might not be large enough.

Based on the findings of the largest well-designed population-based study (Adelborg et al., 2017) and the new 2SMR-based causal analysis (Duan et al., 2022), we hypothesized that the reported HRs and ORs (being less than one) are valid and reproducible when tested using large independent cohorts. For further in-depth investigation, we used a new and large GWAS on AD dataset published in Bellenguez et al. (2022) which contains a larger sample size (487,511 individuals of European ancestry). Importantly, Duan et al. (2022) used nine SNPs as IVs in their study, however, there is evidence of heterogeneity among these nine SNPs in the larger GWAS dataset (Cochran Q test *p* < 5%). Thus, we identified a set of five stable SNPs that had homogeneous effect on the 2SMR test using a leave-one-out sensitivity analysis. Using these five SNPs and the summary data from the GWAS on AD dataset newly published in Bellenguez et al. (2022), we found a statistically significant causal relationship between genetically predicted HF and a lower risk of late-onset AD in the European-descent population. Moreover, the power of our study using the GWAS on AD dataset published in Bellenguez et al. (2022) was compared with the Duan et al. (2022) dataset. Our dataset reached 97.9% power to detect a causal effect, whereas the power based on the dataset used by Duan et al. (2022) is relatively low (36.1%). Thus, we found statistically significant OR <1which is consistent in direction to those ORs published in Duan et al. (2022) and also consistent in the directions of HRs for long-term follow-up obtained in the Danish nationwide population-based cohort study (Adelborg et al., 2017). It should be pointed out that neither Duan et al. (2022) nor Adelborg et al. (2017) have discussed the specific directions of their estimated HRs or ORs. Duan et al. (2022) were more concerned about the potential lack of statistical significance of their findings than the specific directions and implications of their estimated ORs while Adelborg et al. (2017) were more focused on the significant elevated risk of HF on vascular dementia and other non-AD dementias and did not discuss the potential that HF might be associated with lowered risk of AD. Importantly, we have formally tested the hypothesis that genetically predicted HF and a lowered risk of late-onset AD using the 2SMR analysis and our results are statistically significant. Moreover, the fact that ORs <1 indicates a significant causal association between genetically predicted HF and a lowered risk of late-onset AD. We speculate the observed significant ORs (being less than one) reflect the effect of HF-related medications, survival bias, and other effects. In general, in the studied populations, heart failure is commonly treated with HF-related medications and many of them have been reported to have protective effects on cognitive decline and AD (Barthold et al., 2020; Poly et al., 2020; Nguyen et al., 2022; Olmastroni et al., 2022). Also, it is estimated that AD pathology might begin 20 years before the clinical diagnosis of AD. Thus, patients of HF may have less chance to develop late-onset AD because HF is a leading cause of short survival among elderly persons in western populations.

As a limitation of the current study, all individuals included in this study were of European ancestry, limiting the applicability of our finding to more diverse ethnic populations. Additionally, the HF treatment information is not available for analysis in the datasets we analyzed, so we were not able to find more relevant datasets and thus not able to further assess the potential impact of the treatments of HF on late-onset AD. In addition, there is a lack of gender-specific GWAS summary data in the available database. Thus, we are not able to assess whether the causal association between genetically predicted HF and AD is strong in men or women. In future studies, when gender-specific data are available, it is worth further investigating whether the relationship between HF and AD is significantly different between men and women. Importantly, identification and characterization of genetic and other biological mechanisms shared by HF and AD underlying the identified causal relationships would be highly desired but are beyond the scope of this current 2SMR analysis based on limited summary data from existing publications.

In summary, the current 2SMR study indicates a causal association between genetically predicted HF and a lower risk of late-onset AD in the persons with European-ancestry. One of the possible reasons of the observed causal association between genetically predicted HF and a lower risk of late-onset AD might be related to the treatment effects of HF-medications. Currently, AD is the sixth top cause of death without an effective treatment or prevention. AD is a major healthcare and financial burden in the United States and other aging populations. Thus, it is of potentially significant clinical and public health impact to further investigate whether some combinations of HF treatments/medications can be used to develop an effective preventive procedure for late-onset AD. In particular, future identifications and characterizations of genetic factors that underlie the HF-related medications and evidences of protective effect on cognitive decline and AD would be of interest. Further studies including possible clinical trials and new laboratory studies are warranted to conduct in-depth investigation of the statistically significant causal relationship of genetically predicted HF and a lowered risk of AD discovered in the current study.

## Data Availability

The original contributions presented in the study are included in the article/Supplementary Material, further inquiries can be directed to the corresponding author.
