# Supplementary material for: Heart failure and late-onset Alzheimer’s disease: A Mendelian randomization study
Source: Front Genet. 2022 Nov 29;13:1015674. doi: 10.3389/fgene.2022.1015674 (PMC9745072; doi:10.3389/fgene.2022.1015674)
Supplement: Supplementary file 1 [file DataSheet1.PDF]

Supplementary Material

1 Supplementary Figures and Tables

1.1 Supplementary Tables

Supplementary Table 1. Candidate SNPs as instrumental variables (IVs) for MR analysis of the causal effect of HF on AD. These instrumental variables are the same as Table 4 of (Duan et al., 2022).

SNP	Chr	Pos	Gene	A1	A2	EAF	Beta	SE	pval	F
rs11745324	5	137012171	KLHL3	A	G	0.23	-0.053	0.010	2.34E-08	30.89
rs1510226	6	160816409	SLC22A3	C	T	0.01	0.162	0.029	1.27E-08	32.31
rs17042102	4	111668626	PITX2, FAM241A	A	G	0.12	0.110	0.012	5.71E-20	83.10
rs17617337	10	121426884	BAG3	T	C	0.22	-0.056	0.010	3.65E-09	34.87
rs4135240	6	36647680	CDKN1A	C	T	0.34	-0.049	0.008	6.84E-09	33.47
rs55730499	6	161005610	LPA	T	C	0.07	0.106	0.016	1.83E-11	45.41
rs56094641	16	53806453	FTO	G	A	0.42	0.045	0.008	1.21E-08	32.21
rs600038	9	136151806	ABO, SURF1	C	T	0.21	0.057	0.010	3.68E-09	35.13
rs660240	1	109817838	CELSR2	C	T	0.21	0.061	0.010	3.25E-10	39.68

SNP, single-nucleotide polymorphism; Chr, chromosome; Pos, position; A1, effect allele; A2, non-effect allele; EAF, Effect allele frequency; Beta, beta estimate for the association of SNP with HF; SE, standard error; pval, p-value from the meta-analysis of HF.

Supplementary Table 2. 2SMR estimates the causality between HF and AD using the selected 5 SNPs. The summary result used Shah et al. (2020) GWAS on HF and Kunkle et al. (2019) GWAS on AD summary datasets.

Methods	nSNPs	OR (95% CI)	P-value	Q pval	Intercept pval
IVW	5	0.774 (0.586,1.021)	0.070	0.963	
MR Egger	5	0.923 (0.411, 2.074)	0.859	0.942	0.680
Weighted median	5	0.745 (0.527, 1.052)	0.094		
Weighted mode	5	0.729 (0.464, 1.147)	0.243		

nSNPs, number of SNPs; OR, odds ratio; CI, confidence interval; Q pval, p-value of the Cochran Q test; IVW, inverse-variance weighted; Intercept pval, p value of MR-Egger intercept test.

1.2 Supplementary Figures

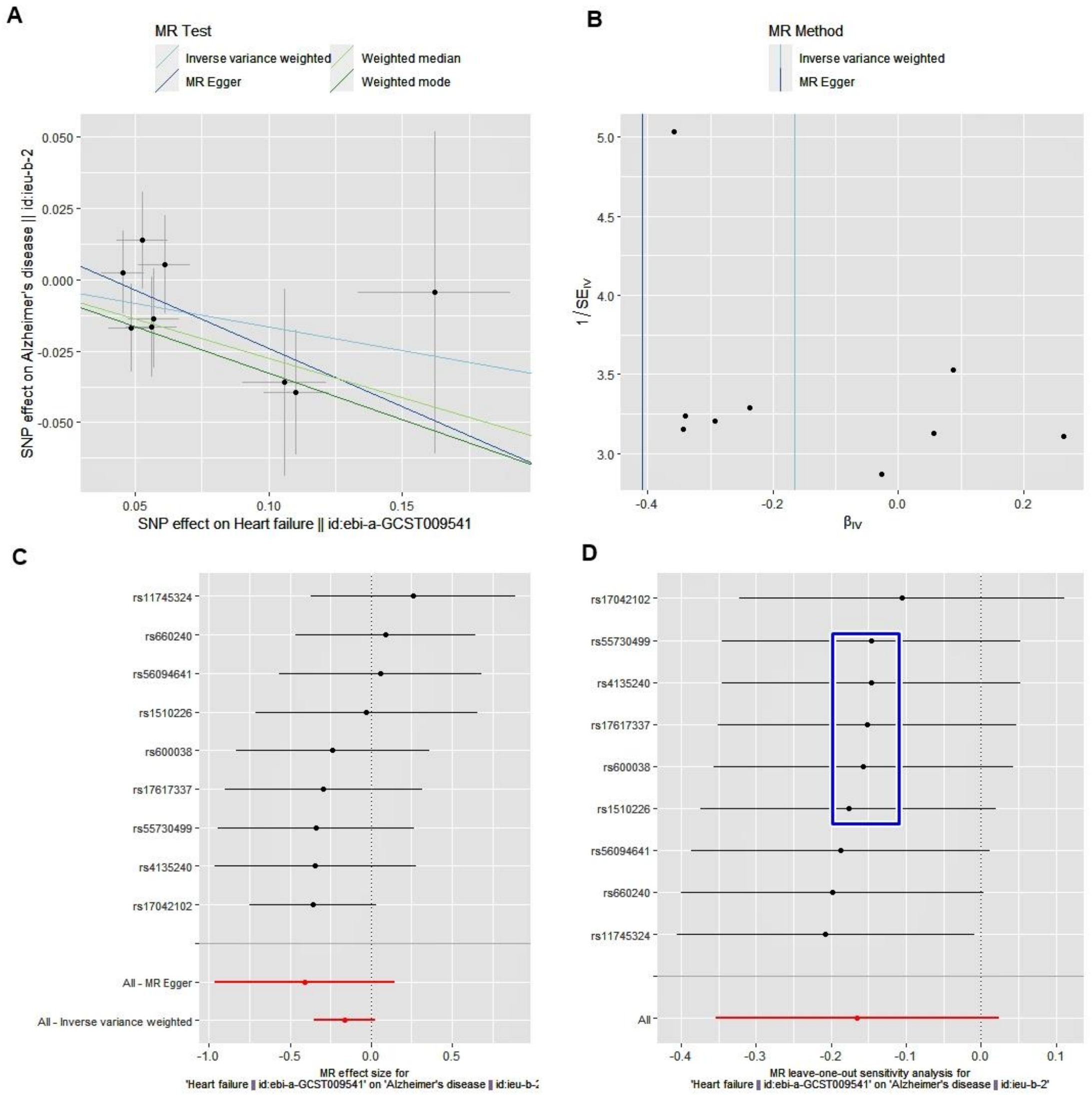

Supplementary Figure 1. 2SMR analysis of the causal association between HF and AD using Kunkle et al. (2019) AD GWAS dataset. This result is the same as those Duan et al. (2022). (A) Scatter plot. The colored lines represent the different methods of causal estimate, and the horizontal and vertical lines crossing at each point show 95% CI for each polymorphism. (B) Funnel plot. The vertical line shows a causal estimate using all SNPs combined into a single instrument for two different methods. (C) Forest plot. Each black dot represents the MR estimate of each SNP, and the horizontal line represents the 95% CI. The red points show a combined causal estimate using all SNPs in a single instrument, including the 2SMR estimates of IVW and MR-Egger. (D) Leave-one-out sensitivity analysis. Each black dot represents the result of MR-IVW excluding that particular SNP, and the associated horizontal line depict 95% CI. The red dot depicts the IVW estimate using all SNPs. The blue box shows a subset of stable SNPs on the 2SMR test.

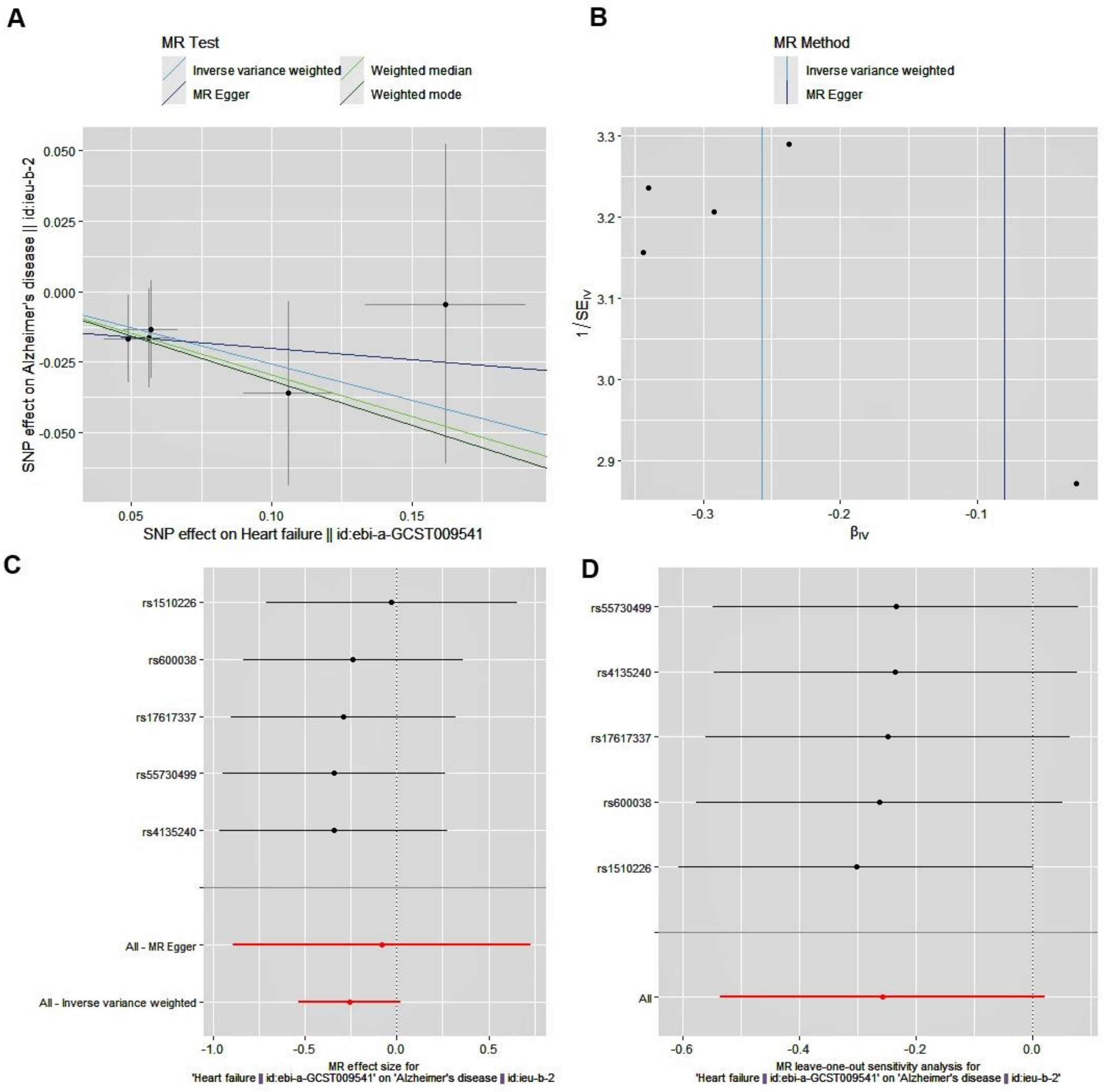

Supplementary Figure 2. 2SMR analysis of the causal association between HF and AD using selected 5 instrumental variables and Kunkle et al. (2019) AD GWAS dataset. (A) Scatter plot. The colored lines represent the different methods of causal estimate, and the horizontal and vertical lines crossing at each point show 95% CI for each polymorphism. (B) Funnel plot. The vertical line shows a causal estimate using all SNPs combined into a single instrument for two different methods. (C) Forest plot. Each black dot represents the MR estimate of each SNP, and the horizontal line represents the 95% CI. The red points show a combined causal estimate using all SNPs in a single instrument, including the 2SMR estimates of IVW and MR-Egger. (D) Leave-one-out sensitivity analysis. Each black dot represents the result of MR-IVW excluding that particular SNP, and the associated horizontal line depict 95% CI. The red dot depicts the IVW estimate using all SNPs.

2 References

Duan, C., Shi, J., Yuan, G., Shou, X., Chen, T., Zhu, X., et al. (2022). Causal association between heart failure and Alzheimer's disease: A two-sample bidirectional mendelian randomization study. *Frontiers in Genetics* 12.

Kunkle, B.W., Grenier-Boley, B., Sims, R., Bis, J.C., Damotte, V., Naj, A.C., et al. (2019). Genetic meta-analysis of diagnosed Alzheimer's disease identifies new risk loci and implicates Aβ, tau, immunity and lipid processing. *Nature genetics* 51(3)**,** 414-430.

Shah, S., Henry, A., Roselli, C., Lin, H., Sveinbjörnsson, G., Fatemifar, G., et al. (2020). Genome-wide association and Mendelian randomisation analysis provide insights into the pathogenesis of heart failure. *Nature communications* 11(1)**,** 1-12.
